# Relationship between serum PTH, potassium (K+), calcium (Ca2+), blood phosphate (PO4), parathyroid hormone (PTH), albumin (ALB) levels and orthostatic hypotension in hemodialysis patients

**DOI:** 10.5937/jomb0-56622

**Published:** 2025-10-28

**Authors:** Shasha Hu, Yuling Chen, Qin Yang, Jun Wen, Aimin Zhong

**Affiliations:** 1 Jiangxi Provincial People's Hospital, The First Affiliated Hospital of Nanchang Medical College, Nanchang, Jiangxi Province, China

**Keywords:** serum PTH, potassium (K+), calcium (Ca2+), blood phosphate (PO4), parathyroid hormone (PTH), albumin (ALB), hemodialysis, orthostatic hypotension, diabetes, serum albumin, independent risk factors, serumski PTH, kalijum (K+), kalcijum (Ca2+), fosfat u krvi (PO4), paratireoidni hormon (PTH), albumin (ALB), hemodijaliza, ortostatska hipotenzija, dijabetes, serumski albumin, nezavisni faktori rizika

## Abstract

**Background:**

Orthostatic hypotension (OH) in maintenance hemodialysis (MHD) patients is a frequent chronic complication. OH may lead to inadequate dialysis, cardiovascular complications, and death. This study explored the relationship between OH and various factors, including serum levels of parathyroid hormone (PTH), potassium (K+), calcium (Ca2+), blood phosphate (PO4), albumin (ALB) levels and Orthostatic Hypotension in Hemodialysis Patients.

**Methods:**

121 MHD patients were enrolled, and their clinical data were acquired. They were categorised into a control (Ctrl) group (normal patients) and an observation (Obs) group (OH patients) based on the diagnostic criteria for OH. Differences in clinical data between patients in different groups were compared, and binary logistic regression (BLR) analysis was performed to assess contributing factors.

**Results:**

Among 121 MHD patients, 40 (33.06%) experienced OH. Comparative analysis demonstrated that patients in the OH group were significantly older, had higher supine systolic blood pressure, increased prevalence of diabetes, and elevated PTH levels, with concomitantly lower blood pressure responses at 1 and 3 minutes after standing, as well as reduced ALB and triglyceride levels (P&lt;0.05). Binary logistic regression analysis further identified advanced age and comorbid diabetes as independent risk factors, whereas higher ALB levels were independently protective against OH.

**Conclusions:**

This study confirms a relatively high incidence of OH in MHD patients and underscores that advanced age, diabetes, and low serum albumin levels are significant independent predictors of OH. These findings suggest that early identification and targeted intervention in high-risk patients could improve hemodialysis outcomes and reduce cardiovascular complications.

## Abstract (sr)

**Uvod:**

Ortostatska hipotenzija (OH) kod pacijenata na bolničkoj hemodijalizi (MHD) predstavlja čestu komplikaciju. OH može dovesti do neadekvatne dijalize, kardiovaskularnih komplikacija i smrtnog ishoda. Ova studija ispituje odnos izme|u OH i različitih faktora, uključujući nivoe paratireoidnog hormona (PTH), kalijuma (K+), kalcijuma (Ca2 +), fosfata u krvi (PO4) i albumina (ALB) kod pacijenata na hemodijalizi.

**Metode:**

U istraživanje je uključen 121 pacijent na MHD, čiji su klinički podaci prikupljeni i analizirani. Na osnovu dijagnostičkih kriterijuma za OH, pacijenti su svrstani u kontrolnu grupu (Ctrl) (pacijenti bez OH) i posmatranu grupu (Obs) (pacijenti sa OH). Upoređene su razlike u kliničkim podacima između grupa, a binarna logistička regresija (BLR) je korišćena za procenu faktora koji doprinose nastanku OH.

**Rezultati:**

Od 121 pacijenta na MHD, 40 (33,06%) je imalo OH. Komparativna analiza pokazala je da su pacijenti sa OH bili značajno stariji, imali su viši sistolni krvni pritisak u ležećem položaju, veću učestalost dijabetesa i povišene nivoe PTH, uz istovremeno niži krvni pritisak nakon 1 i 3 minuta stajanja, kao i smanjene nivoe ALB i triglicerida (P&lt;0,05). Binarna logistička regresiona analiza pokazala je da su poodmakla starost i komorbiditet sa dijabetesom nezavisni faktori rizika, dok su viši nivoi ALB bili zaštitni faktor u odnosu na OH.&nbsp;

**Zaključak:**

Ova studija potvrđuje relativno visoku učestalost OH kod pacijenata na MHD i ukazuje na to da su poodmakla starost, dijabetes i niski nivoi serumskog albumina značajni nezavisni prediktori OH. Ovi nalazi sugerišu da rana identifikacija i ciljane intervencije kod pacijenata sa visokim rizikom mogu da poboljšaju ishode hemodijalize i smanje kardiovaskularne komplikacije.

## Introduction

Maintenance hemodialysis (MDH) is a transitional approach that utilises blood dialysis or peritoneal dialysis to save patients’ lives and extend the lifespan of end-stage renal disease patients [1]. The mortality rate among end-stage renal disease patients is significantly higher than that of the general population, and MDH is a common renal replacement therapy for such patients. However, various chronic complications often occur during MDH treatment, with hypotension being the most common [2] [3] [4]. Orthostatic hypotension (OH) is defined as a clinical syndrome in which a patient experiences a drop in systolic blood pressure (SBP) of at least 20 mmHg or a drop in diastolic pressure of at least 10 mmHg within 3 minutes of transitioning from a supine to an upright position, with or without various symptoms of hypoperfusion [5]. OH patients typically exhibit symptoms when changing from a supine to an upright position, including dizziness, blurred vision, palpitations, and so on. Some patients may experience OH without any conscious symptoms, leading to its being overlooked. OH increases the risk of patients falling, fainting, and experiencing accidental injuries [6]. Research has shown that elevated levels of norepinephrine in the bloodstream, decreased sensitivity of beta-adrenergic receptors, and reduced nitric oxide content in vascular endothelium are all factors that can lead to abnormalities in the patient’s autonomic nervous system, thus triggering the occurrence of OH [7]. Additionally, when patients have comorbid conditions such as hypertension, diabetes, and high cholesterol, the use of multiple medications can also increase the risk of OH [8]. Therefore, effective prevention of hypotension during blood dialysis treatment is paramount.

The primary objective of this study was to investigate the relationship between OH and several clinical and biochemical parameters – including serum levels of PTH, K^+^, Ca_2_^+^, PO_4_, ALB, and additional relevant indicators – in MHD patients. By identifying independent risk factors for OH, this study aims to contribute to the development of effective preventive strategies and clinical interventions for high-risk hemodialysis patients.

## Materials and methods

### Basic information

121 patients who received hemodialysis treatment at ** Hospital from June 2022 to June 2023 were selected, including 78 males and 43 females. They were 21–71 years old, averaging (49.26±13.92) years. The body mass index (BMI) ranged from 13.50 to 38.90 kg/m^2^, with an average value of (22.11±3.97) kg/m^2^. The duration of dialysis was 7–181 months, which was averaged as (69.49±45.74) months. Patients enrolled had to satisfy all the following conditions: 1) age over 18 years, 2) MDH treatment duration exceeding 3 months, 3) weekly hemo dialysis duration between 8 to 12 hours, and 4) ability to stand independently and cooperate with BP measurements in both supine and upright positions. The patients with any of the following conditions had to be excluded: 1) acute renal failure, 2) previous kidney transplantation, 3) peritoneal dialysis, 4) recent major surgical procedures, 5) severe cardiovascular or cerebrovascular complications preventing independent standing, 6) advanced malignant tumors, and 7) pregnant women. All enrolled patients had provided informed consent.

This study was conducted at Jiangxi Provincial People’s Hospital, Nanchang, China. The study protocol was approved by the Jiangxi Provincial People’s Hospital Ethics Committee (Ethical Code: JP-NMC-2022-045).

### Methodologies

### Data acquisition

General patient information, including age, gender, hemodialysis duration, BMI, and primary disease, was collected. Data on the BP of patients at 1 minute and 3 minutes in both the supine and upright positions, dry weight, and ultrafiltration were also collected. Blood samples for laboratory tests were obtained by collecting fasting venous blood on dialysis days.

### Diagnosis of OH and patient groups

The d OH during dialysis was diagnosed in accordance with the standards set by the Kidney Disease Outcomes Quality Initiative (K/DOQI) guidelines [9]. The HEM-1020 electronic blood pressure monitor (Omron, Japan) was utilised to measure the upper arm BP of patients. Before measurement, patients had to rest quietly for 510 minutes. After the rest of 5–10 minutes after dialysis, the blood pressure of the upper arm of the non-fistula hand was measured 3 times in the supine position, the measurement interval of 1 minute, and the average value was recorded. Subsequently, patients were instructed to change from a supine to an upright position, and BP measurements of the right upper arm were taken again at 1 minute and 3 minutes after the change; each measurement was repeated three times with a 1-minute interval, and the average value was recorded. The diagnosis of OH was made when a patient’s SBP suddenly dropped by at least 20 mmHg or when the mean arterial pressure dropped by at least 10 mmHg, with this occurrence happening three or more times over a continuous three-month period and being related to clinical events requiring intervention. Additionally, if patients experienced symptoms such as cramps, nausea, vomiting, dizziness, headache, chest tightness, abdominal pain, blurred vision, or loss of consciousness during dialysis, with at least one adverse reaction, the diagnosis of OH was confirmed. Based on the occurrence of OH, the 121 patients were assigned to a Ctrl group, consisting of 81 patients who did not experience OH during dialysis, and an Obs group, comprising 40 patients who experienced OH during dialysis.

### Laboratory data

Fasting venous blood was acquired and subjected to various laboratory tests at the Hospital’s clinical laboratory, encompassing measurements of blood potassium (K^+^), blood calcium (Ca_2_^+^), blood phosphate (PO_4_), total cholesterol (TC), triglycerides (TG), parathyroid hormone (PTH), albumin (ALB), and hemoglobin (Hb) levels.

### Methods for statistical analysis

Data were organised and analysed using SPSS 19.0 software. Continuous variables that followed a normal distribution were expressed as (x̄±s), and comparisons were made using independent sample t-tests. Binary variables were presented as counts or percentages, and comparisons were performed adopting the χ^2^ test. The analysis of factors influencing OH during dialysis was implemented employing BLR analysis. All statistical tests were two-tailed. *P*<0.05 indicated statistical significance.

## Results

### Basic information about patients

121 hemodialysis patients were enrolled, encompassing 40 who experienced OH, accounting for 33.06% of the total. Subsequently, a comparison of general patient characteristics between the Ctrl group and the Obs group was conducted, as outlined in Table 1. It was evident that differences between Ctrl and Obs group patients were not obvious in terms of gender, BMI, hemodialysis duration, dry weight, ultrafiltration, and dry weight-to-ultrafiltration ratio (DW-to-U) (*P*>0.05). However, patients in the Obs group were obviously older, with considerable differences from the Ctrl group (*P*<0.05).

**Table 1:** Basic information of patients (n=121).

Item		Ctrl group	Obs group	* t/χ^2^*	P
Sample size		81	40		
Age (years old)		49.26±13.92	55.98±10.54	-2.691	0.008
Gender (cases/%)	Males	51/62.96	27/67.50	0.624	0.689
	Females	30/37.04	13/32.50		
BMI (kg/m^2^)		22.11±3.97	22.18±2.74	-0.093	0.926
Hemodialysis duration		69.49±45.74	63.53±35.74	0.723	0.471
Dry weight (kg)		60.66±14.47	61.28±10.18	-0.243	0.808
Ultrafiltration (L)		3.37±0.65	3.05±0.81	0.343	0.732
DW-to-U (%)		5.72±1.19	5.03±1.20	0.314	0.682

### Statistics of primary disease of patients

**Figure 1:**
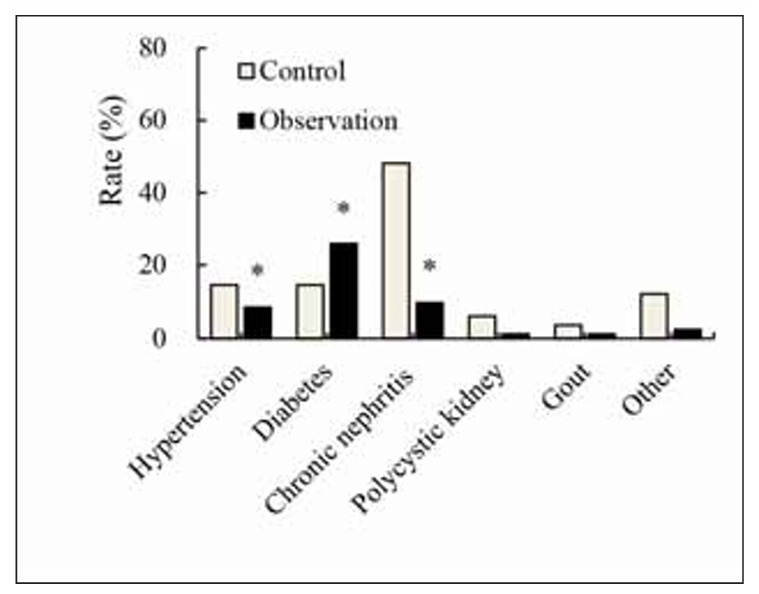
Statistics of primary disease of patients (n=121). Note: * suggested a remarkable difference with P<0.05 to the Ctrl group.

A comparison of the differences in primary diseases between the Ctrl and Obs groups was illustrated in Figure 1. In the Ctrl group, there were 12 cases of hypertension (14.81%), 12 cases of diabetes (14.81%), 39 cases of chronic nephritis (48.15%), 5 cases of polycystic kidney disease (6.17%), 3 cases of gout (3.70%), and 10 cases of other primary diseases (12.35%). In contrast, 7 cases of hypertension (8.64%), 21 cases of diabetes (25.93%), 8 cases of chronic nephritis (9.88%), 1 case of polycystic kidney disease (1.23%), 1 case of gout (1.23%), and 2 cases of other primary diseases (2.47%) were observed in Obs group. It was observed that the incidence of hypertension and chronic nephritis in the Obs group was lower in contrast to that in the Ctrl group, showing obvious differences (*P*<0.05). However, the Obs group exhibited a higher incidence of diabetes, demonstrating a visible difference relative to the Ctrl group (*P*<0.05).

### Changes in BP-related indicators

**Figure 2:**
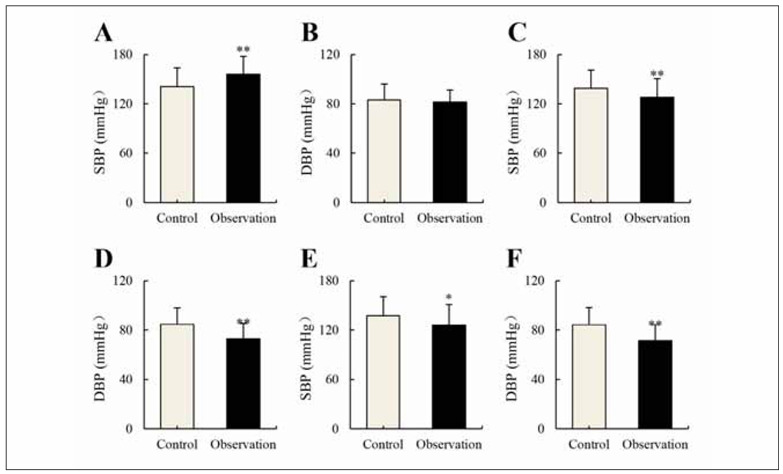
Values of supine BP in various groups (n=121) (A): supine SBP; (B): supine DBP; (C) SBP at 1 minute after standing; (D) DBP at 1 minute after standing; (E) SBP at 3 minutes after standing; and (F) DBP at 3 minutes after standing. Note: * and ** suggested a remarkable difference with P<0.05 and P<0.01 in the Ctrl group, respectively.

Differences in BP-related indicators between patients in varying groups were compared in Figure 2. It demonstrated no visible differences in supine DBP for patients in Ctrl and Obs groups (*P*>0.05). However, patients in the Obs group experienced sharply higher supine SBP relative to those in the Ctrl group, while SBP and DBP, at 1 minute and 3 minutes after standing, were greatly lower in the Obs group. These comparisons revealed no remarkable differences between the Obs and Ctrl groups (*P*<0.05).

### Changes in serum-relevant parameters

**Figure 3:**
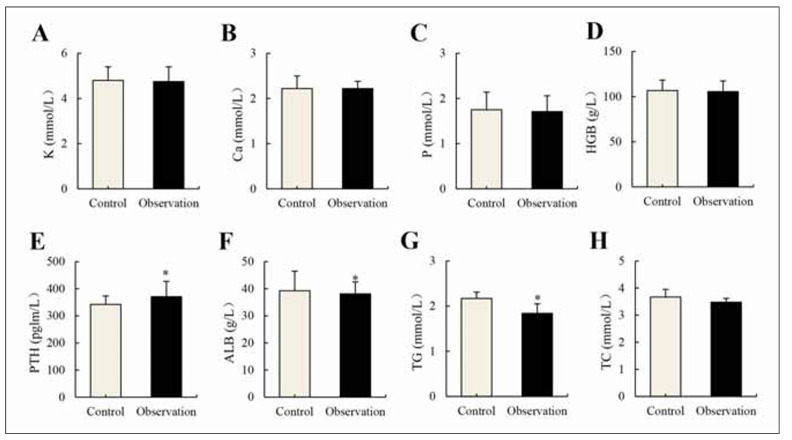
Changes in serum-relevant parameters (*n*=121). (A): potassium; (B): calcium; (C): phosphate; (D): Hb; (E): PTH; (F): ALB; (G): TG; and (H): TC. Note: * suggested a remarkable difference with P<0.05 to the Ctrl group.

Figure 3 depicts differences in serum-related indicators between Ctrl and Obs groups. It was evident that patients in distinct groups exhibited no obvious difference in serum potassium, calcium, phosphate, Hb, and TC levels (*P*>0.05). However, patients in the Obs group presented higher PTH as well as lower ALB and TC in comparison to the Ctrl group (*P*<0.05).

### Results of BLR analysis for influencing factors of OH

To further explore the factors contributing to OH in our study population, we conducted a multifactorial binary logistic regression (BLR) analysis. This analysis included the variables that showed significant differences between the Ctrl and Obs groups in the previous comparisons. The results of the BLR analysis are presented in Table 2.

**Table 2:** Results of BLR analysis for factors influencing OH.

Indicator	B	SE	OR	95% CI	P
Age	0.522	0.083	1.408	1.123~1.679	0.047
Diabetes	0.735	0.115	2.149	1.530~4.387	0.018
PTH	0.534	0.029	0.981	0.544~1.905	0.360
ALB	-0.491	0.074	1.073	0.786~1.590	0.029
TC	-0.083	0.162	0.718	0.524~1.124	0.286

Our analysis revealed that advanced age and comorbid diabetes were independent risk factors for developing OH during dialysis. For each year increase in age, the odds of experiencing OH increased by 40.8% (OR=1.408, 95% CI: 1.123–1.679, P=0.047). Patients with diabetes had more than twice the odds of having OH compared to those without diabetes (OR=2.149, 95% CI: 1.530–4.387, P=0.018). Conversely, a higher serum albumin (ALB) level was identified as a protective factor against OH. Each unit increase in ALB was associated with a 7.3% reduction in the odds of OH (OR=1.073, 95% CI: 0.786-1.590, P=0.029). Although PTH and TC levels differed significantly between the groups in the univariate analysis, they were not found to be independent predictors of OH in the BLR analysis.

The BLR analysis provides quantitative evidence of the impact of each variable on the risk of OH. Specifically, the OR of 1.408 indicates that with each additional year of age, the likelihood of OH increases by 40.8%, underscoring the vulnerability of older patients. Similarly, the OR of 2.149 for diabetes suggests that diabetic patients are at a significantly higher risk, potentially due to underlying autonomic dysfunction. As reflected in the regression model, the protective effect of ALB emphasises the role of nutritional status and plasma oncotic pressure in maintaining hemodynamic stability during dialysis. These findings are consistent with previous literature linking aging, diabetes, and hypoalbuminemia with OH in MHD patients.

## Discussion

Hypotension during hemodialysis is a multifactorial and complex complication that is particularly prevalent among the elderly, often resulting in fainting, falls, and cardiovascular events, which can markedly affect patient outcomes [10] [11]. In our study, a 33.06% incidence of OH was observed among 121 MHD patients, a finding that is consistent with previous reports indicating an incidence range of 11% to 39.2% in elderly populations [12] [13]. This high incidence underscores the substantial risk of OH in this vulnerable group and highlights the need for rigorous monitoring and intervention.

There is no universal standard for the diagnosis of OH. While the American Autonomic Society and the American Academy of Neurology recommend a diagnostic threshold based solely on a drop in blood pressure – specifically, a decline in systolic BP of at least 20 mmHg and/or diastolic BP of at least 10 mmHg within 3 minutes of standing [14] – our study employed the K/DOQI guidelines. These guidelines integrate quantitative blood pressure measurements and the presence of clinical symptoms, thereby providing a more comprehensive assessment. This dual approach likely enhances the detection of clinically significant OH, even in cases where the blood pressure drop is less pronounced.

Through binary logistic regression analysis, this research revealed that age influences OH occurrence in hemodialysis patients and is an independent risk factor. This may be due to the fact that as patients age, vascular elasticity and function become compromised, making them more susceptible to OH when changing positions [15] [16]. Therefore, it emphasises the importance of paying particular attention to elderly hemodialysis patients to prevent OH and the high-risk events it can lead to, including falls.

Gannon et al. found that the probability of OH occurrence in diabetes patients was 22%, compared to 13% in non-diabetes patients. A logistic regression model confirmed a remarkable relationship between blood glucose levels and the incidence of OH [17]. Beretta et al. [18] also confirmed that the probability of diabetes patients experiencing concurrent OH is higher, and the risk of falling and death for diabetes patients with OH is 2.70 times and 1.54 times higher than that of non-diabetes patients. In this work, BLR analysis revealed that comorbid diabetes is an independent risk factor for the development of OH in hemodialysis patients. This may be due to the fact that chronic underlying diseases can affect a patient’s cardiovascular regulatory function, leading to autonomic nervous system dysfunction. Furthermore, the reduction in sympathetic nervous system response and decreased vascular responsiveness to sympathetic stimulation in the later stages of hemodialysis may contribute to hypotension [19] [20] [21]. Therefore, it is recommended for dialysis patients to use sugar-free or low-sugar dialysate whenever possible during the dialysis process.

Serum markers, particularly serum albumin (ALB), also play a critical role in the pathophysiology of OH. ALB, a liver-synthesised protein crucial for maintaining plasma colloid osmotic pressure, serves as an important indicator of a patient’s nutritional status [22] [23]. Ueda et al. [24] found that the occurrence of ALB leakage in hemodialysis patients can impact the levels of glycated ALB, which has a significant correlation with blood glucose levels. Zhao et al. [25] constructed a decision tree model to identify high-risk individuals for injurious falls in the elderly population, and they identified OH and serum ALB levels as IRFs for injurious falls in elderly patients. Hypoalbuminemia is a risk factor for early death in MHD patients [26]. Tang et al. [27] demonstrated that early ALB levels can reflect the nutritional and chronic inflammatory status of hemodialysis patients and serve as an independent predictor of mortality in hemodialysis patients [27]. Maiwall et al. [28] confirmed that the administration of 20% albumin to patients with hypotension caused by factors like sepsis can lead to a more rapid improvement in hemodynamics and lactate clearance, with a reduced need for dialysis. The BLR analysis in this work revealed that low ALB levels were IRFs for the progression of OH in hemodialysis patients. This may be attributed to the fact that low ALB levels can affect the plasma colloid osmotic pressure of a patient, leading to a decrease in effective circulating blood volume and factors like reduced vascular refilling [29]. Therefore, maintaining higher serum ALB levels during hemo dialysis is recommended as it acts as a protective factor in preventing OH.

In addition to intrinsic patient factors, extrinsic factors such as rapid ultrafiltration and the biocompatibility of the dialysis membrane can further exacerbate hypotensive episodes. Rapid fluid removal can compromise blood volume and cardiac output [30], while the use of certain antihypertensive or sedative medications, in conjunction with the biocompatibility of the dialysis membrane, may also contribute to the onset of hypotension [31].

Overall, our findings corroborate previous research regarding the high incidence of OH in MHD patients and elucidate the critical roles of advanced age, diabetes, and low serum ALB in its pathogenesis. The utilisation of the K/DOQI guidelines, which incorporate both hemodynamic and symptomatic criteria, may offer a more sensitive and clinically relevant diagnostic approach compared to criteria based solely on numerical blood pressure changes.

Furthermore, recent investigations have expanded our understanding of the biochemical and inflammatory mechanisms underlying OH. For example, studies have reported that elevated levels of inflammatory cytokines, such as interleukin-6 and tumour necrosis factor-alpha, are associated with endothelial dysfunction and may predispose patients to OH during hemodialysis [32]. Additionally, research focusing on oxidative stress markers, including increased malondialdehyde and decreased antioxidant enzyme activity, has suggested a contributory role in the hemodynamic instability observed in these patients [33] [34]. Moreover, emerging clinical evidence supports the notion that individualised ultrafiltration protocols, which take into account patient-specific fluid status and vascular compliance, can significantly reduce the frequency and severity of OH episodes [35]. These insights highlight the multifactorial nature of OH and underscore the importance of integrating both inflammatory and oxidative stress biomarkers into future predictive models for better risk stratification and management of MHD patients.

## Conclusion

This study revealed a significant prevalence of orthostatic hypotension (OH) in maintenance hemodialysis (MHD) patients, with 33.06% of the study population experiencing this complication. We identified advanced age, comorbid diabetes, and low serum albumin levels as independent risk factors for OH. These findings emphasise the need for vigilant monitoring and early intervention in high-risk MHD patients to prevent falls, syncope, and cardiovascular complications. Implementing targeted interventions, such as patient education, medication adjustments, and close monitoring of fluid balance, may help reduce the incidence and adverse outcomes associated with OH.

While this study provides valuable insights into OH in MHD patients, limitations include the small sample size and short follow-up period. Further research with larger cohorts and longer follow-ups is needed to validate these findings and explore the effectiveness of various interventions in preventing and managing OH. This study serves as a foundation for future research and clinical practice to improve the care and outcomes of MHD patients susceptible to OH.

## Dodatak

### Conflict of interest statement

All the authors declare that they have no conflict of interest in this work.
